# Patient-Reported Outcomes after Intensity-Modulated Proton Therapy for Oropharynx Cancer

**DOI:** 10.14338/IJPT-20-00081.1

**Published:** 2021-06-25

**Authors:** Houda Bahig, Brandon G. Gunn, Adam S. Garden, Rong Ye, Kate Hutcheson, David I. Rosenthal, Jack Phan, Clifton D. Fuller, William H. Morrison, Jay Paul Reddy, Sweet Ping Ng, Neil D. Gross, Erich M. Sturgis, Renata Ferrarotto, Maura Gillison, Steven J. Frank

**Affiliations:** 1 The University of Texas MD Anderson Cancer Center, Houston, TX, USA; 2 Centre Hospitalier de l'Université de Montréal, Montreal, Canada; 3 Peter MacCallum Cancer Center, Melbourne, Australia

**Keywords:** intensity-modulated proton therapy, oropharynx cancer, head and neck, quality of life, FACT-HN

## Abstract

**Purpose:**

To report patient-reported outcomes (PROs) derived from the Functional Assessment of Cancer Therapy-Head and Neck (FACT-HN) tool, in patients with oropharynx cancer (OPC) treated with intensity-modulated proton therapy (IMPT) in the context of first-course irradiation.

**Materials and Methods:**

Patients with locally advanced OPC treated with radical IMPT between 2011 and 2018 were included in a prospective registry. FACT-HN scores were measured serially during and 24 months following IMPT. PRO changes in the FACT-HN scores over time were assessed with mixed-model analysis.

**Results:**

Fifty-seven patients met inclusion criteria. Median age was 60 years (range, 41-84), and 91% had human papillomavirus-associated disease. In total, 28% received induction chemotherapy and 68% had concurrent chemotherapy. Compliance to FACT-HN questionnaire completion was 59%, 48%, and 42% at 6, 12, and 24 months after treatment, respectively. The mean FACT-General (G), FACT-Total, and FACT-Trial Outcome Index (TOI) score changes were statistically and clinically significant relative to baseline from week 3 of treatment up to week 2 after treatment. Nadir was reached at week 6 of treatment for all scores, with maximum scores dropping by 15%, 20%, and 39% compared to baseline for FACT-G, FACT-Total, and FACT-TOI, respectively. Subdomain scores of physical well-being, functional well-being, and head and neck additional concerns decreased from baseline during treatment and returned to baseline at week 4 after treatment.

**Conclusions:**

IMPT was associated with a favorable PRO trajectory, characterized by an acute decline followed by rapid recovery to baseline. This study establishes the expected acute, subacute, and chronic trajectory of PROs for patients undergoing IMPT for OPC.

## Introduction

Owing to its high-dose conformity and reduced toxicity, notably reduced rates of xerostomia compared to 3D conformal radiation therapy [1], use of intensity-modulated photon radiation therapy (IMRT) has been widely adopted in head and neck irradiation. Nevertheless, given the large treatment volume along with the proximity to structures such as the oral cavity, pharyngeal constrictors, and salivary glands, there remains significant treatment morbidity and deterioration of patients' quality of life from head and neck irradiation [2, 3]. As the oropharynx cancer (OPC) population is now largely composed of middle-aged patients with favorable-prognosis human papillomavirus (HPV)-associated OPC [4–6], it has become imperative to further improve quality of life in these patients, most of whom are predicted to live for many years.

The intrinsic dosimetric properties of proton beam therapy (PBT), characterized by the Bragg peak and sharp lateral dose gradient, make PBT a promising strategy to improve tolerability of large-field head and neck irradiation [7]. In particular, the recent development of intensity-modulated proton therapy (IMPT) offers greater potential for dose optimization in the treatment of complex volumes of the head and neck region [8]. The dosimetric advantage of IMPT over IMRT has been well demonstrated, with studies showing reduction of dose to the oral cavity, swallowing structures, and salivary glands [9–11]. Although preliminary clinical outcome data support excellent cancer control outcomes and favorable toxicity profile of IMPT in OPC [12], results from an ongoing multi-institutional randomized phase III trial comparing outcomes of IMPT to IMRT in OPC treated with concurrent chemoradiation are eagerly awaited [13].

Patient-reported outcomes (PROs) provide critical insight into patients' symptoms and function after therapy [14, 15]. A definition of expected PRO trajectory is critical for adequate value-based assessment of IMPT and effective comparison with IMRT. Using the MD Anderson Symptom Inventory for Head and Neck Cancer (MDASI-HN) instrument, only 1 study has reported PROs after IMPT in OPC to date [16]. In a matched-cohort analysis, Sio et al [16] reported significantly lower subacute symptom burden after IMPT for OPC as compared to IMRT. The Functional Assessment of Cancer Therapy-Head and Neck (FACT-HN) questionnaire is another validated instrument used widely to report longitudinal PRO changes after HNC treatment [17]. In the current study, we report PROs derived from the FACT-HN tool in patients with OPC treated with IMPT in the context of first-course irradiation.

## Materials and Methods

### Patient Population and Treatment Characteristics

Patients with OPC treated with PBT were enrolled in a prospective institutional study (PA11-0803) at MD Anderson Cancer Center between 2011 and 2018. This study was approved by our institutional review board; all enrolled patients provided informed consent. For the current analysis, the following inclusion criteria were used: (1) age ≥ 18 years, (2) biopsy-proven squamous cell carcinoma (SCC) of the oropharynx, (3) treatment with curative-intent scanning-beam IMPT at our institution, and (4) having completed baseline and at least 1 posttreatment FACT-HN questionnaire. Patients with non-SCC histology, prior irradiation of the head and neck, or evidence of distant metastases were excluded. Patients treated with induction chemotherapy, concurrent systemic therapy, or adjuvant radiation therapy were included. All cases were presented to a multidisciplinary tumor board. Selected patients with T4 or N2c-N3 stage (as per AJCC 7th edition [18]) were offered induction chemotherapy, taking into consideration patients' age, comorbidities, and symptoms, after discussion at tumor board. Treatment immobilization method as well as dosimetric and planning details are described elsewhere [19].

### PRO Symptom Burden Assessment

PROs were measured by using the validated and multidimensional FACT-HN instrument, version 4 [20, 21], a thoroughly tested instrument [22], to establish a benchmark for the expected PRO trajectory of patients undergoing IMPT for OPC. The FACT-HN is a 38-item questionnaire measuring quality of life related to head and neck cancer (HNC) treatment. The maximum score is 144 and reflects the best possible quality of life. It contains the following 5 subdomains: physical well-being (PWB), social/family well-being (SWB), emotional well-being (EWB), functional well-being (FWB), and additional head and neck cancer concerns (HNCC). Three summary scores are then derived: (1) the FACT-general (FACT-G) score (score range, 0-108), which sums the first 4 subdomain scores (PWB, SWB, EWB, and FWB); (2) the FACT-Total score (range, 0-144), which sums all 5 subdomains; and (3) the FACT-Trial Outcome Index score FACT-TOI (score range, 0-92), which sums the PWB, FWB, and HNCC subdomains. Missing data were handled in accordance with the FACIT Administration and Scoring Guidelines (at www.facit.org). FACT-HN completion schedule was as follows: at baseline; every week during the course of IMPT; at 2, 4, 6, 8, and 10 weeks after IMPT; and at 6, 12, and 24 months after IMPT. The baseline FACT-HN questionnaire was completed on the day of the planning clinic, typically within 1 week before IMPT start.

### Statistics

Follow-up duration was defined as the time from the date of diagnosis to the date of last follow-up or death. Follow-up was calculated by using the reverse Kaplan-Meier method. Disease-free survival was estimated by using the Kaplan-Meier method, with disease-free survival events including death, primary failure, regional failure, and distant metastasis. Compliance was defined as the number of FACT-HN questionnaires completed by patients over the number of disease-free survivors at a given time point. A longitudinal analysis consisting of repeated measures mixed-effects models was used. All reported *P* values from multilevel analysis were 2 sided, and levels <.05 were considered statistically significant. Differences were considered clinically significant if the change from baseline (absolute value) was greater than half of the baseline standard deviation, based on a previously described distribution-based method [23, 24]. Statistical analyses were done with SAS software (release 9.4; SAS Institute, Cary, North Carolina).

## Results

### Patient Population and Disease-Free Survival

**Figure 1.**
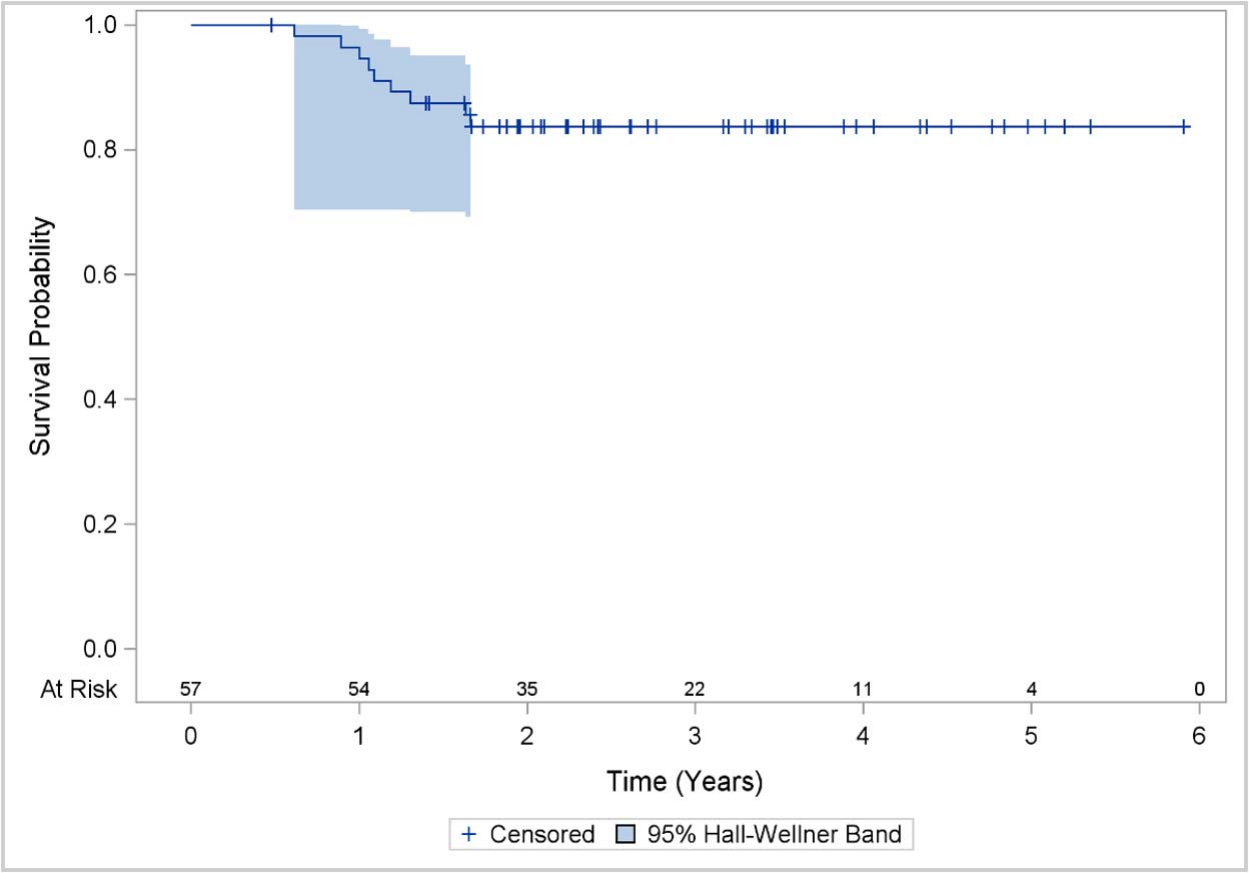
Disease-free survival as a function of time.

Among 125 patients with OPC included in this prospective registry, only 57 met inclusion criteria, with 68 patients excluded for the following reasons: non-SCC histology, re-irradiation, palliative irradiation, or lack of follow-up FACT-HN questionnaire completion. Patients and treatment characteristics are described in Table 1. In summary, median age was 60 years (range, 41-84), 86% of patients were male, 91% had HPV-associated OPC, and 49% of patients had no smoking history. Overall disease stage was II, III, IVA, and IVB in 2% (1 patient), 14%, 79%, and 5% of patients, respectively (as per AJCC 7th edition [18]). In total, 42% had concurrent chemo-IMPT, 28% had induction chemotherapy followed by IMPT ± chemotherapy, 23% had IMPT alone, and 7% had TORS followed by concurrent chemo-IMPT. At a median follow-up time of 2.8 years (95% CI, 2.2-3.5), there were 4 (7%) primary failures, 1 (2%) neck failure, and 4 (7%) distant failures. Disease-free survival rates at 1 year and 2 years were 95% and 84%, respectively (Figure 1).

**Table 1.** Patients and treatment characteristics.

**Parameter**	**Value, n (%)**
Sex	
Female	8 (14)
Male	49 (86)
Age, mean (range), y	60 (41-84)
Overall stage per AJCC 7th edition [18]	
II	1 (2)
III	8 (14)
IVA	45 (79)
IVB	3 (5)
Overall stage per AJCC 8th edition [25]	
I	35 (61)
II	11 (19)
III	6 (11)
IVA	4 (7)
IVB	1 (2)
T status per AJCC 7th edition [18]	
T1	20 (35)
T2	25 (44)
T3	4 (7)
T4a	6 (11)
T4b	2 (4)
T status per AJCC 8th edition [25]	
T1	20 (35)
T2	23 (40)
T3	4 (7)
T4	9 (16)
T4a	1 (2)
N status per AJCC 7th edition [18]	
N0	3 (5)
N1	8 (14)
N2a	6 (11)
N2b	29 (51)
N2c	9 (16)
N3	2 (4)
N status per AJCC 8th edition [25]	
N0	3 (5)
N1	41 (72)
N2	8 (14)
N2a	1 (2)
N2b	1 (2)
N2c	1 (2)
N3	2 (4)
HPV/p16 status	
Negative	2 (4)
Positive	52 (91)
Unknown	3 (5)
Smoking history	
Current smoker	3 (5)
Never smoked	28 (49)
Past smoker	26 (46)
Pack-year history (among smokers), mean (range)	27 (1-96)
Treatment course	
Concurrent chemo-IMPT	24 (42)
Induction + chemo-IMPT	11 (19)
Induction + IMPT	5 (9)
IMPT	13 (23)
TORS + chemo-IMPT	4 (7)

**Abbreviations:** AJCC, American Joint Committee on Cancer Staging Manual; IMPT, intensity-modulated proton therapy; TORS, trans-oral robotic surgery.

### FACT-HN Outcomes

**Figure 2.**
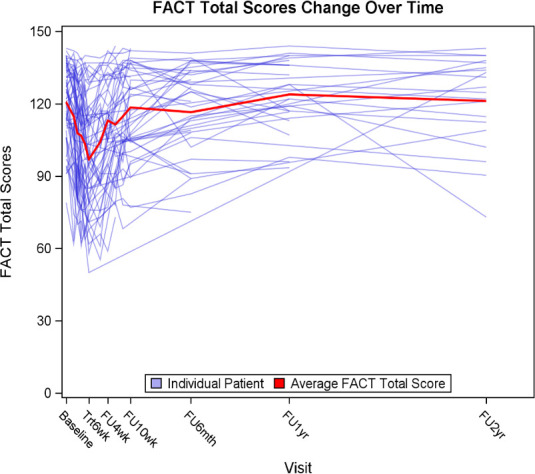
Individual patient's FACT-Total scores change over time. Abbreviation: FACT, Functional Assessment of Cancer Therapy.

**Figure 3.**
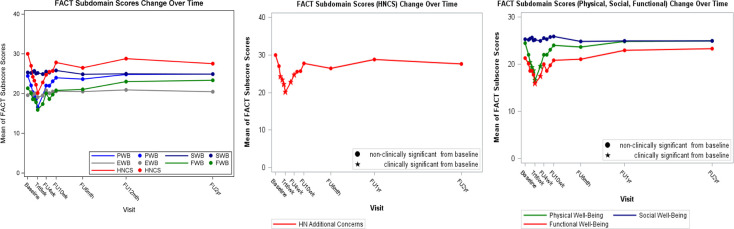
FACT subdomain scores change over time. Abbreviation: FACT, Functional Assessment of Cancer Therapy.

**Figure 4.**
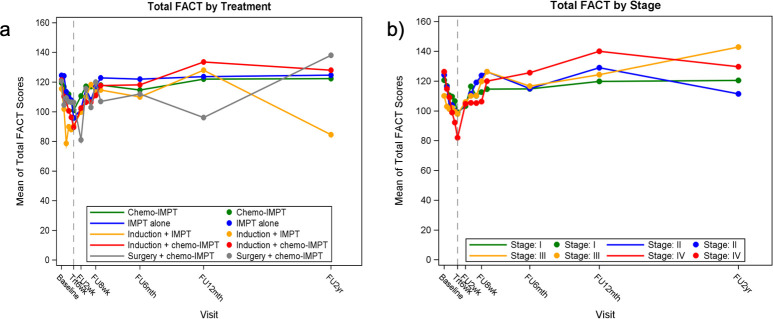
FACT-Total score by (a) treatment type and (b) disease stage. Abbreviation: FACT, Functional Assessment of Cancer Therapy.

**Figure 5.**
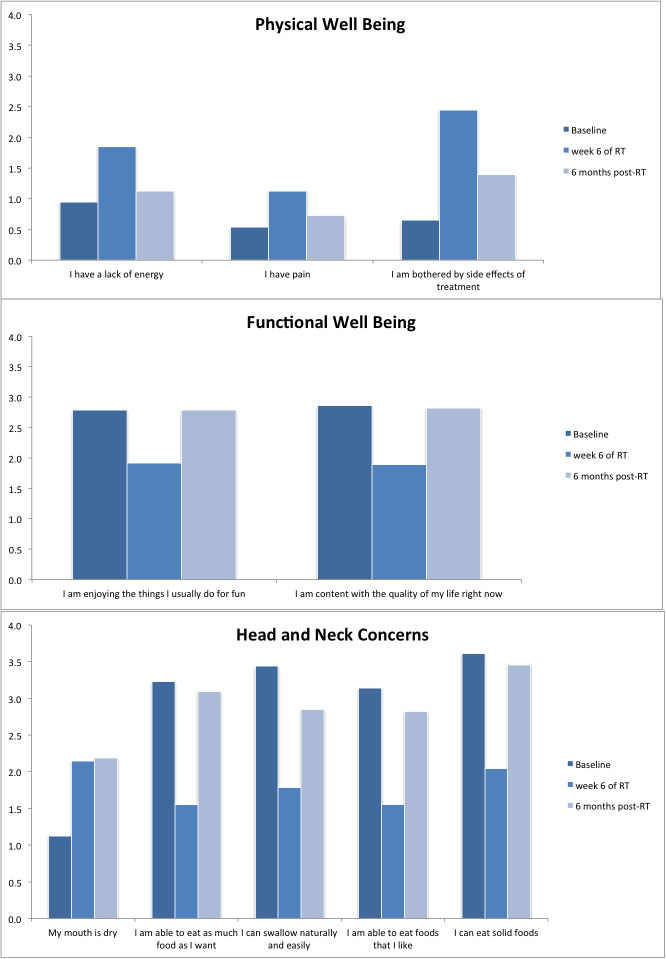
Worst scoring items in the subdomains of physical well being, functional well being, and head and neck concerns.

Compliance rates were 100%, 82%, 59%, 48%, and 42% at baseline, at 6 weeks of treatment, and at 6, 12, and 24 months after treatment, respectively. The FACT-HN summary and subdomain scores at each visit as well as the changes from baseline at each visit are summarized in Table 2 and **Supplementary Material 1**, respectively. Baseline FACT-G, FACT-Total, and FACT-TOI scores were 91 (maximum score, 108), 121 (maximum score, 144) and 76 (maximum score, 92). The mean FACT-G and FACT-Total score changes were significantly worse (statistically and clinically) compared to baseline from week 3 of treatment up to week 2 after treatment, and the mean FACT-TOI score also dropped at week 3 of treatment but returned to baseline at week 4 after treatment. Nadir was reached at week 6 of treatment for all scores, with maximum scores dropping by 14, 24, and 23 points, compared to baseline, for FACT-G, FACT-Total, and FACT-TOI, respectively (**Supplementary Material 1**). Figure 2 shows FACT-Total scores change over time for all patients. Similarly, subdomain scores of PWB and HNCC decreased from baseline at week 3 of treatment (mean drop of 4 and 6 points, respectively), reached a nadir at week 6 of treatment (mean drop of 8 and 10 points, respectively), and rapidly returned to baseline at week 4 after treatment (Figure 3). Figure 4 and **Supplementary Material 2** show FACT-Total score by stage and treatment type over time. Functional well-being deterioration was clinically and statistically significant from week 6 of treatment to week 2 after treatment. Worst scoring items in the subdomains of PWB, FWB, and HNCC are presented in Figure 5. There were no differences in EWB and SWB subdomains across time.

**Table 2.** FACT summary scores and subdomain scores by visit.

**Visit**	**FACT scores**							**FACT subdomain scores**										
	**FACT-G**			**FACT-Total**		**FACT-TOI**		**PWB**		**SWB**		**EWB**		**FWB**			**HNCC**	
	**N**	**Mean**	**SD**	**Mean**	**SD**	**Mean**	**SD**	**Mean**	**SD**	**Mean**	**SD**	**Mean**	**SD**	**N**	**Mean**	**SD**	**Mean**	**SD**
Trt1wk	57	91	13	121	16	76	12	24	4	25	5	20	3	57	21	6	30	5
Trt2wk	52	88	15	115	20	69	16	22	5	25	5	20	3	53	20	6	27	6
Trt3wk	51	84	15	108	20	63	18	20	5	25	4	20	3	52	19	7	24	8
Trt4wk	52	83	16	107	22	61	19	19	7	26	4	20	3	52	19	6	23	8
Trt5wk	50	81	16	103	22	58	18	19	6	25	4	20	3	50	18	7	22	8
Trt6wk	47	77	17	97	23	53	19	17	7	25	4	19	4	47	16	7	20	8
Fu2wk	42	81	18	104	24	60	20	20	7	25	4	20	4	42	17	7	23	8
Fu4wk	34	88	16	113	21	67	17	22	5	26	4	21	3	34	20	7	25	7
Fu6wk	36	86	15	111	20	66	17	22	5	25	4	20	3	36	19	7	25	7
Fu8wk	37	89	15	115	20	69	17	23	4	26	4	20	4	37	20	7	26	7
Fu10wk	32	91	13	118	18	72	15	24	4	26	4	21	3	33	21	6	28	6
Fu6mo	33	90	15	117	18	71	14	24	4	25	4	21	3	33	21	6	26	6
Fu12mo	24	94	12	124	15	77	12	25	3	25	4	21	3	25	23	5	29	6
Fu2yr	20	94	14	121	19	76	15	25	4	25	6	20	3	20	23	6	28	7

**Abbreviations:** FACT, Functional Assessment of Cancer Therapy; G, General; TOI, Trial Outcome Index; PWB, physical well-being; SWB, social/family well-being; EWB, emotional well-being; FWB, functional well-being; HNCC, head and neck cancer concerns; N, number; Trt, treatment; wk, week; Fu, follow-up; mo, month; yr, year.

## Discussion

PROs have been increasingly integrated into physician-patient shared decision-making [26] and are now considered a critical outcome measure, both from a clinical patient-centered perspective and from a socioeconomic perspective, as an indicator of indirect costs of treatment morbidity [27]. To our knowledge, this is the largest study assessing quality of life outcomes after IMPT for OPC. We report acute declines in PROs during treatment in the FACT summary scores, physical and functional well-being scores, and head and neck cancer concerns scores. This decline reached a nadir at week 6 of treatment, with all scores rapidly returning to baseline values by week 4 after treatment. This study establishes the expected acute, subacute, and chronic trajectory of PROs among patients undergoing IMPT for OPC.

The overall trajectory of our cohort, characterized by a fast recovery to high baseline scores, is suggestive of a highly favorable toxicity profile. The PROs we report in this study compare well with modern cohorts of PROs reported in the era of IMRT. Ringash et al [28] reported FACT-HN outcomes at 6 and 12 months after hyperfractionated accelerated IMRT for locally advanced HNC. Although the authors did not report acute and subacute PROs, they found an improvement in functional and emotional scores compared to baseline and no detrimental effect in all other scores at 6 months after treatment. In another study of 54 patients treated with concurrent chemo-IMRT using a de-escalated nodal dose of 36 Gy, Maguire et al [29] assessed longitudinal changes in FACT-HN scores and reported a recovery to baseline scores at only 6 months after treatment. The minimal subacute symptom burden we report is also supported by a previous study by our group comparing concurrent chemo-IMPT (35 patients) to concurrent chemo-IMRT (46 patients) in OPC, in a nonrandomized fashion [16]. In the latter study, although no differences in MSADI-HN symptom burden scores were noted in the acute and chronic recovery phases, symptom burden was found to be lower among patients treated with IMPT during the subacute recovery phase (defined as 3 months after treatment end) than among patients treated with IMRT.

Our study has several limitations. First, the study does not provide any head-to-head comparison with PROs from IMRT. Results from the MD Anderson randomized phase III trial of IMRT versus IMPT for oropharynx cancer (NCT01893307) are awaited and will shed light on the comparative effectiveness of the 2 treatment modalities. The heterogeneity of patients (inclusion of HPV-associated and HPV-negative OPC, smokers and nonsmokers) and treatment approaches (inclusion of patients treated with induction chemotherapy, concurrent chemotherapy, and TORS) is another limitation. In fact, it has previously been demonstrated that systemic treatment is associated with higher symptom burden [30, 31] and one can expect varying recovery of the PRO trajectory, based on the intensity of the treatment combination. Similarly, previous findings from the Trans Tasman Radiation Oncology Group (TROG) 02.02 study comparing FACT-HN scores for p16-positive versus p16-negative OPC treated with chemoradiation have shown a distinct PRO trajectory in p16-positive OPC, characterized by a more abrupt decline from baseline to 2 months after treatment, followed by a faster recovery by 6 months, compared to p16-negative OPC [32]. The high preponderance of HPV-associated OPC in our cohort, the young patient age (median, 60 years), and the high overall baseline score can certainly explain in part the observed capacity for rapid posttreatment recovery. Finally, 2 other limitations intrinsic to PRO studies require consideration. The first is that of decreasing compliance over time, with only about 1 in 2 patients having completed the FACT-HN questionnaire at 1 year. The challenge of maintaining PRO questionnaire completion in head and neck cancer studies has previously been described [17, 28] and in fact our compliance rates are fairly similar to those of previous prospective reports in similar patient populations, with compliance rates varying between 45% and 60% at 6 to 12 months [28, 33–36]. This may have biased our results favorably in the event that noncompliant patients happen to have worse PRO trajectory. This common and recognized problem of missing long-term data in quality of life studies limits the external validity of these studies [17] and warrants cautious data interpretation. The second intrinsic limitation is that of suggestion bias, whereby patients who are aware they are receiving PBT would expect lower toxicity, a limitation that only patient blinding (highly challenging, if not impossible) could address.

In conclusion, as assessed by the FACT-HN instrument, IMPT for locally advanced OPC was associated with a highly favorable PRO trajectory characterized by a recovery to high baseline scores within 1 month of treatment completion, therefore suggesting minimal subacute and chronic symptom burden. While further data from the current MD Anderson randomized trial comparing the clinical effectiveness of IMPT to that of IMRT in OPC are awaited, results of this study are reassuring in regard to early and late treatment tolerability, and add to the growing data on the value of PBT in the management of OPC.
