# Supplementary material for: Patient-Reported Outcomes after Intensity-Modulated Proton Therapy for Oropharynx Cancer
Source: Int J Part Ther. 2021 Jun 25;8(1):213–22. doi: 10.14338/IJPT-20-00081.1 (PMC8270092; doi:10.14338/IJPT-20-00081.1)
Supplement: Supplementary file 1 [file ijpt-08-01-23_s01.docx]

***Supplementary Material 1. FACT-HN Summary and Subdomain Scores Changes from Baseline at Each Visit***

| **Score** | **Visit** | **N** | **Mean of Change** | **SD** | **P-value** |
| --- | --- | --- | --- | --- | --- |
|  |  |  | **(FU - Baseline)** |  |  |
| **FACT-G** | **Trt2wk** | 52 | -3 | 9 | 0.999 |
|  | **Trt3wk** | 51 | -7 | 11 | 0.431 |
|  | **Trt4wk** | 52 | -8 | 12 | 0.200 |
|  | **Trt5wk** | **50** | **-10** | **11** | **0.028** |
|  | **Trt6wk** | **47** | **-14** | **14** | **0.000** |
|  | **Fu2wk** | **42** | **-11** | **14** | **0.029** |
|  | **Fu4wk** | 34 | -5 | 13 | 0.684 |
|  | **Fu6wk** | 36 | -5 | 12 | 0.730 |
|  | **Fu8wk** | 37 | -2 | 11 | 0.991 |
|  | **Fu10wk** | 32 | 0 | 11 | 0.999 |
|  | **Fu6mth** | 33 | 2 | 10 | 0.996 |
|  | **Fu12mth** | 24 | 3 | 11 | 0.908 |
|  | **Fu2yr** | 20 | 3 | 13 | 0.913 |
| **FACT-TOI** | **Trt2wk** | 53 | -6 | 12 | 0.410 |
|  | **Trt3wk** | **52** | **-13** | **13** | **0.000** |
|  | **Trt4wk** | **52** | **-15** | **15** | **0.000** |
|  | **Trt5wk** | **49** | **-17** | **14** | **0.000** |
|  | **Trt6wk** | **47** | **-23** | **18** | **0.000** |
|  | **Fu2wk** | **42** | **-17** | **18** | **0.000** |
|  | **Fu4wk** | **34** | **-11** | **16** | **0.042** |
|  | **Fu6wk** | 36 | -10 | 16 | 0.070 |
|  | **Fu8wk** | 37 | -7 | 14 | 0.365 |
|  | **Fu10wk** | 33 | -2 | 15 | 0.922 |
|  | **Fu6mth** | 33 | -1 | 12 | 0.988 |
|  | **Fu12mth** | 25 | 1 | 12 | 0.975 |
|  | **Fu2yr** | 20 | -1 | 13 | 0.965 |
| **FACT-TOTAL** | **Trt2wk** | 52 | -6 | 13 | 0.901 |
|  | **Trt3wk** | **51** | **-13** | **15** | **0.012** |
|  | **Trt4wk** | **52** | **-15** | **17** | **0.004** |
|  | **Trt5wk** | **49** | **-17** | **16** | **0.000** |
|  | **Trt6wk** | **47** | **-23** | **20** | **0.000** |
|  | **Fu2wk** | **42** | **-18** | **20** | **0.001** |
|  | **Fu4wk** | 34 | -11 | 19 | 0.196 |
|  | **Fu6wk** | 36 | -10 | 18 | 0.284 |
|  | **Fu8wk** | 37 | -6 | 16 | 0.804 |
|  | **Fu10wk** | 32 | -1 | 16 | 0.986 |
|  | **Fu6mth** | 33 | -1 | 13 | 0.998 |
|  | **Fu12mth** | 24 | 2 | 15 | 0.961 |
|  | **Fu2yr** | 20 | -1 | 16 | 0.974 |
| **EWB** | **Trt2wk** | 53 | 1 | 3 | 0.993 |
|  | **Trt3wk** | 52 | 0 | 3 | 1.000 |
|  | **Trt4wk** | 52 | 0 | 3 | 0.999 |
|  | **Trt5wk** | 50 | 0 | 3 | 0.997 |
|  | **Trt6wk** | 47 | -1 | 4 | 0.985 |
|  | **Fu2wk** | 42 | 0 | 4 | 0.979 |
|  | **Fu4wk** | 34 | 1 | 3 | 0.951 |
|  | **Fu6wk** | 36 | 1 | 3 | 0.981 |
|  | **Fu8wk** | 37 | 1 | 3 | 0.976 |
|  | **Fu10wk** | 33 | 1 | 3 | 0.921 |
|  | **Fu6mth** | 33 | 1 | 3 | 0.947 |
|  | **Fu12mth** | 24 | 2 | 3 | 0.555 |
|  | **Fu2yr** | 20 | 1 | 4 | 0.761 |
| **FWB** | **Trt2wk** | 53 | -1 | 5 | 0.997 |
|  | **Trt3wk** | 52 | -3 | 6 | 0.552 |
|  | **Trt4wk** | 52 | -3 | 5 | 0.515 |
|  | **Trt5wk** | 50 | -4 | 5 | 0.194 |
|  | **Trt6wk** | **47** | **-5** | **7** | **0.006** |
|  | **Fu2wk** | **42** | **-5** | **6** | **0.024** |
|  | **Fu4wk** | 34 | -3 | 6 | 0.609 |
|  | **Fu6wk** | 36 | -3 | 6 | 0.480 |
|  | **Fu8wk** | 37 | -2 | 6 | 0.887 |
|  | **Fu10wk** | 33 | -1 | 6 | 0.983 |
|  | **Fu6mth** | 33 | 2 | 5 | 0.946 |
|  | **Fu12mth** | 25 | 2 | 5 | 0.857 |
|  | **Fu2yr** | 20 | 2 | 6 | 0.700 |
| **HNCS** | **Trt2wk** | 54 | -3 | 5 | 0.266 |
|  | **Trt3wk** | **52** | **-6** | **7** | **0.000** |
|  | **Trt4wk** | **52** | **-7** | **7** | **0.000** |
|  | **Trt5wk** | **49** | **-8** | **7** | **0.000** |
|  | **Trt6wk** | **47** | **-10** | **8** | **0.000** |
|  | **Fu2wk** | **42** | **-7** | **9** | **0.000** |
|  | **Fu4wk** | **34** | **-5** | **7** | **0.018** |
|  | **Fu6wk** | 36 | -4 | 7 | 0.070 |
|  | **Fu8wk** | 37 | -4 | 7 | 0.151 |
|  | **Fu10wk** | 34 | -2 | 6 | 0.784 |
|  | **Fu6mth** | 33 | -3 | 6 | 0.499 |
|  | **Fu12mth** | 25 | -1 | 6 | 0.825 |
|  | **Fu2yr** | 20 | -3 | 5 | 0.357 |
| **PWB** | **Trt2wk** | 53 | -2 | 4 | 0.228 |
|  | **Trt3wk** | **52** | **-4** | **5** | **0.000** |
|  | **Trt4wk** | **52** | **-5** | **6** | **0.000** |
|  | **Trt5wk** | **50** | **-6** | **5** | **0.000** |
|  | **Trt6wk** | **47** | **-8** | **6** | **0.000** |
|  | **Fu2wk** | **42** | **-5** | **6** | **0.001** |
|  | **Fu4wk** | 34 | -3 | 6 | 0.107 |
|  | **Fu6wk** | 36 | -3 | 5 | 0.176 |
|  | **Fu8wk** | 37 | -1 | 4 | 0.762 |
|  | **Fu10wk** | 33 | 0 | 4 | 0.991 |
|  | **Fu6mth** | 33 | 0 | 5 | 0.981 |
|  | **Fu12mth** | 25 | 1 | 4 | 0.933 |
|  | **Fu2yr** | 20 | 0 | 5 | 0.939 |
| **SWB** | **Trt2wk** | 53 | 0 | 2 | 1.000 |
|  | **Trt3wk** | 51 | 0 | 4 | 1.000 |
|  | **Trt4wk** | 52 | 0 | 2 | 1.000 |
|  | **Trt5wk** | 50 | -1 | 2 | 1.000 |
|  | **Trt6wk** | 47 | 0 | 4 | 1.000 |
|  | **Fu2wk** | 42 | 0 | 5 | 0.997 |
|  | **Fu4wk** | 34 | 0 | 2 | 1.000 |
|  | **Fu6wk** | 36 | 0 | 3 | 1.000 |
|  | **Fu8wk** | 37 | 0 | 5 | 0.991 |
|  | **Fu10wk** | 33 | 0 | 5 | 0.985 |
|  | **Fu6mth** | 33 | -1 | 3 | 0.999 |
|  | **Fu12mth** | 25 | -1 | 3 | 0.992 |
|  | **Fu2yr** | 20 | -1 | 5 | 0.905 |

***Supplementary Material 2. Summary of Total FACT Score by Stage and Treatment Type***

| **Visit** | **Clinical Stage AJCC 8^th^ Ed** | | | | | | | |
| --- | --- | --- | --- | --- | --- | --- | --- | --- |
|  | **I** | | **II** | | **III** | | **IV** | |
|  | **N** | **Mean** | **N** | **Mean** | **N** | **Mean** | **N** | **Mean** |
| Trt1wk | 35 | 121 | 11 | 124 | 6 | 110 | 5 | 126 |
| Trt2wk | 30 | 117 | 11 | 116 | 6 | 103 | 5 | 115 |
| Trt3wk | 30 | 110 | 10 | 103 | 6 | 101 | 5 | 109 |
| Trt4wk | 31 | 110 | 11 | 104 | 6 | 102 | 4 | 99 |
| Trt5wk | 29 | 107 | 10 | 100 | 6 | 100 | 4 | 92 |
| Trt6wk | 27 | 99 | 9 | 98 | 6 | 98 | 5 | 82 |
| Fu2wk | 24 | 103 | 9 | 105 | 5 | 106 | 4 | 105 |
| Fu4wk | 17 | 116 | 8 | 111 | 5 | 110 | 4 | 106 |
| Fu6wk | 21 | 110 | 7 | 119 | 4 | 110 | 4 | 105 |
| Fu8wk | 22 | 113 | 6 | 124 | 5 | 120 | 4 | 106 |
| Fu10wk | 20 | 115 | 6 | 126 | 3 | 126 | 3 | 120 |
| Fu6mth | 18 | 115 | 5 | 115 | 6 | 117 | 4 | 126 |
| Fu12mth | 12 | 120 | 6 | 129 | 5 | 124 | 1 | 140 |
| Fu2yr | 12 | 120 | 4 | 112 | 1 | 143 | 3 | 130 |

| **Visit** | **Treatment Course** | | | | | | | | | |
| --- | --- | --- | --- | --- | --- | --- | --- | --- | --- | --- |
|  | **Chemo-IMPT** | | **IMPT alone** | | **Induction + IMPT** | | **Induction + chemo-IMPT** | | **Surgery + chemo-IMPT** | |
|  | **N** | **Mean** | **N** | **Mean** | **N** | **Mean** | **N** | **Mean** | **N** | **Mean** |
| Trt1wk | 24 | 119 | 13 | 125 | 5 | 115 | 11 | 121 | 4 | 121 |
| Trt2wk | 22 | 117 | 11 | 124 | 5 | 102 | 11 | 110 | 3 | 105 |
| Trt3wk | 20 | 110 | 12 | 113 | 4 | 79 | 11 | 107 | 4 | 109 |
| Trt4wk | 21 | 112 | 13 | 110 | 5 | 90 | 10 | 101 | 3 | 107 |
| Trt5wk | 20 | 107 | 12 | 108 | 5 | 88 | 9 | 96 | 3 | 106 |
| Trt6wk | 20 | 101 | 10 | 96 | 4 | 91 | 10 | 90 | 3 | 106 |
| Fu2wk | 16 | 111 | 10 | 102 | 4 | 100 | 10 | 102 | 2 | 81 |
| Fu4wk | 13 | 117 | 6 | 116 | 4 | 110 | 9 | 106 | 2 | 116 |
| Fu6wk | 13 | 117 | 8 | 108 | 3 | 118 | 10 | 107 | 2 | 103 |
| Fu8wk | 15 | 115 | 9 | 117 | 3 | 113 | 8 | 111 | 2 | 120 |
| Fu10wk | 14 | 118 | 8 | 123 | 3 | 115 | 6 | 118 | 1 | 107 |
| Fu6mth | 15 | 115 | 7 | 122 | 2 | 110 | 7 | 118 | 2 | 112 |
| Fu12mth | 13 | 122 | 4 | 124 | 1 | 128 | 5 | 134 | 1 | 96 |
| Fu2yr | 7 | 122 | 6 | 125 | 2 | 85 | 4 | 128 | 1 | 138 |
